# A Postural Approach to the Pelvic Diameters of Obstetrics: The Dynamic External Pelvimetry Test

**DOI:** 10.7759/cureus.6111

**Published:** 2019-11-09

**Authors:** Marco Siccardi, Cristina Valle, Fiorenza Di Matteo, Valentina Angius

**Affiliations:** 1 Department of Obstetrics and Gynaecology, San Paolo Hospital, Savona, ITA

**Keywords:** dystocia, labour, childbirth, prevention, obstetrics, postural control, physiology, pelvimetry, anatomy

## Abstract

In recent years, there has been a renewed interest in internal and external pelvimetry, in relation to the diagnosis of dystocia from a "contracted pelvis." Dystocia is still one of the causes of maternal-fetal morbidity and mortality in the world. The main cause is the fetal-pelvic disproportion, of which mechanical dystocia and contracted pelvis are most probably involved. Clinical pelvimetry was the diagnostic method of "contracted pelvis" and still seems to have its place in the clinical obstetric routine. Studies have been conducted in order to measure anatomical diameters and correlate them with operative or vaginal delivery. Some studies have been published regarding the diameters' variation with the shifting of the patient's posture. The positions used in the research for the analysis of changes in pelvis measurements are the same as those used for centuries to assist and promote childbirth.

This technical report is to define a method of measuring changes in classical pelvimetric external diameters in relation to the postural change of the subjects, taking into consideration the needs of the operators, the postural difficulties of pregnant women and the evidence acquired from instrumental research. It aims to propose a dynamic postural method suited to daily practice, according to the directives and principles of the classical external obstetric pelvimetry.

## Introduction

Dystocia is still one of the causes of maternal-fetal morbidity and mortality in the world and is often associated with fetal distress and operative delivery. The main cause is fetal-pelvic disproportion: absolute fetal-pelvic disproportion (FPD) is uncommon, but the performance of cesarean delivery for that indication is common [1]. Mechanical dystocia and contracted pelvis are more likely to be implicated in obstructed labor. In recent years, there has been a renewed interest in studying internal and external obstetrical pelvimetry, concerning the diagnosis of dystocia from the contracted pelvis and operative delivery [2-9]. Lumbopelvic pain before and during pregnancy is a risk factor for mechanical dystocia in labor [10].

Researches have been conducted with instrumental devices (MR, CT, and optoelectronic devices) to evaluate the static diameters of the maternal pelvis with the obstetric outcome [2-5]. They were conducted to measure anatomical static diameters and correlate them to operative or vaginal delivery. Some studies have been published regarding the change of the pelvic diameters with the shifting of the patient's posture: the positions used in the research to analyze changes in pelvis measurements are the same as those used for centuries to assist and promote childbirth [5-7].

Many clinical studies have considered the external diameters of obstetric pelvimetry and the diameters of the sacral rhomboid area to correlate them to the types of delivery, to the obstructed labor risk, and to the diagnosis of the contracted pelvis [8-9].

The instrumental studies indicate a change in the internal diameters of the female pelvis with the posture assumed by the patients, moving from the upright or supine position to the squat (standing, kneeling) position [6-7,11]. The study of Place and colleagues demonstrated that the pelvic incidence, considered a fixed parameter, changed when the subjects varied their pelvic position [12]. It suggests a potential functional motion at the sacroiliac joint during pelvic rotations. It also supports the idea that changing one's posture could lead to a change in pelvic incidence and pelvic tilt and, consequently, to a change in the pelvic space of the birth canal [11-14]. Loading conditions during dynamic labor positions and increased ligament laxity during pregnancy would expand the pelvis [13]. When the internal diameters change, the external ones also change, and vice-versa, and they could be related to the obstetric outcome. Some evidence suggests that the intermediate positions (hands-and-knees position) have a better impact on the dimensions of the diameters of the birth canal than the supine or squat position [11,13]. The dynamic activities and alternative positions are encouraged by midwives during labor and moving may generate greater pelvic mobility than the comparable static posture [14].

Research with optical-electronic instruments evaluates the change of the position in the space of the sensors placed on the external surface of the body [5,11,13-14], but some studies show that external landmarks move with body movement due to the soft tissue artifact that would affect external markers located above those points during movement analysis [15]. The human hand is a very precise and reliable sensor; the hand is one of the most complex and beautiful pieces of natural engineering and it allows us to manipulate small objects with great precision [16]. Furthermore, technical instruments (MR, CT, optoelectronic devices, 3D-reconstruction software) are not available in every part of the world and cannot be used in routine obstetric practice on every single pregnant woman.

The obstetric pelvimeter was an instrument of daily obstetrics practice, presumably present in every obstetric practice in the world, including low-income countries [8-9]. The dynamic examination of the external pelvimetry in shifting positions could be performed in a clinical setting using simple instruments, as any postural biomechanical examination must have a methodology technique and assessment criteria [17], and must be easy to practice and to reproduce.

No technical report has been published so far to define the criteria of a method for measuring the external diameters of obstetrics pelvimetry with the postural change of the subjects, based on evidence acquired by instrumental researches and clinical practice, taking into account the needs of the operators and the difficulties during the movements of pregnant women. This technical report aims to propose an external-pelvimetry postural method suitable for clinical daily routine, even in situations of few economic resources, according to the directives and principles of classical external obstetric pelvimetry.

## Technical report

The present technical report describes the procedure to measure the obstetrical external pelvic diameters in shifting positions and takes into consideration the following methodological topics that are an integral part of the dynamic external pelvimetry (DEP) test.

What to measure

List and description of external pelvic diameters in obstetrics

With which instruments to measure

Description of the modified obstetric pelvimeter of Collin and the "bone-meter kit" (BMK) by Metrica SpA (Milan, Italy).

In which postures to measure

Description of the body positions that can be easily assumed by every pregnant woman, with particular regard to the procedure of shifting from a position to another, in observance of the evidence from previous researches [2-5,11,13-14].

The external pelvic diameters

The diameters of external pelvimetry are divided into the transverse, anteroposterior, and craniocaudal diameters. Some of them have a correspondence with the internal diameters of the pelvic birth canal [9,18]. The transverse diameters are the transversal diameter of the sacral area of Michaelis, the base of the Trillat’s triangle, the ischial inter-tuberosities diameter, the bi-trochanteric diameter, the inter-crestal diameter (iliac crest), the iliac bi-spinous diameter (between the anterior superior iliac spines). The anteroposterior diameter is the external obstetric conjugate (Baudeloque’s diameter). The craniocaudal diameters are the diameter and the longitudinal hemi-diameter of the Michaelis area.

The Michaelis Area

**Figure 1:**
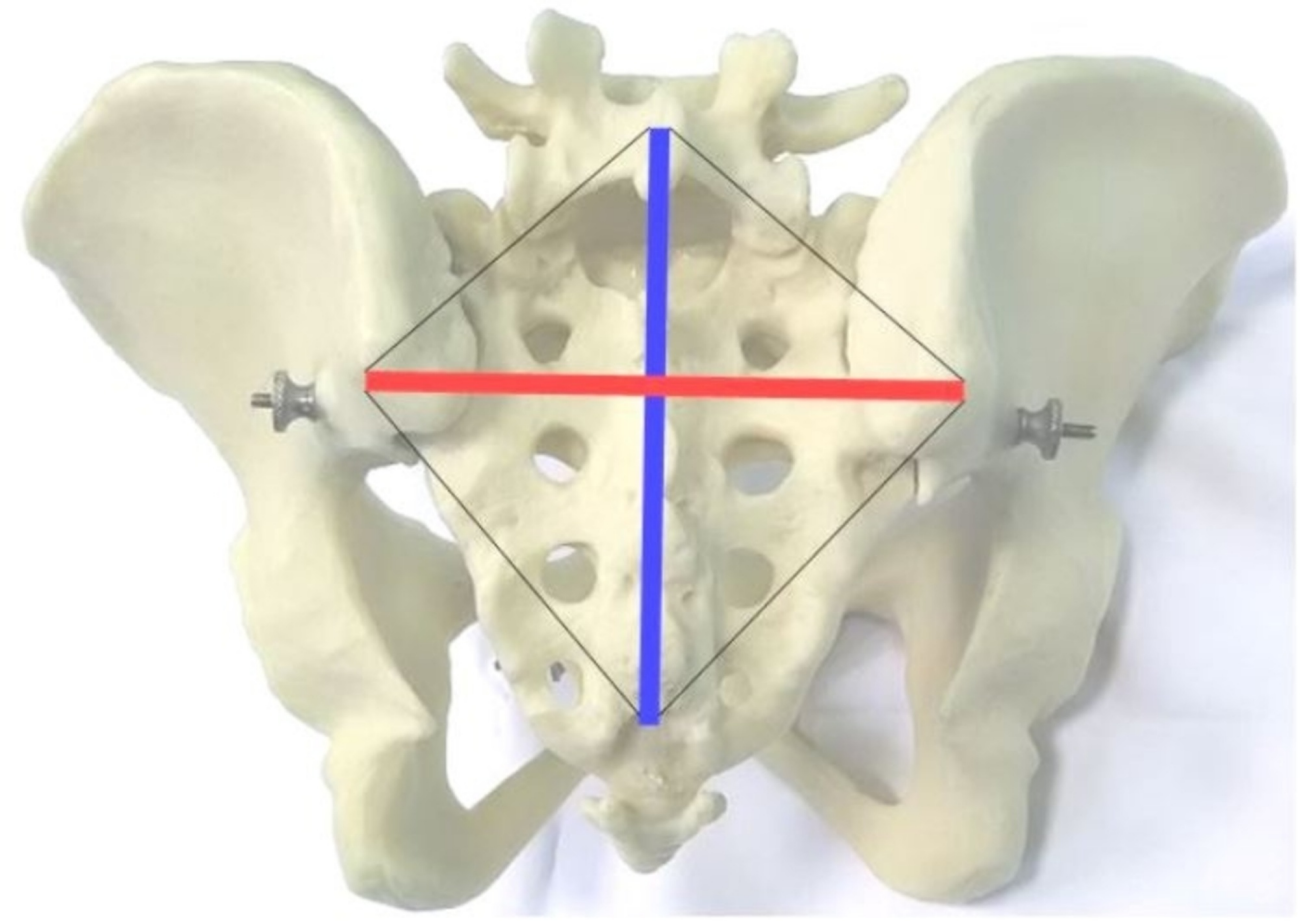
Michaelis' sacral rhomboid area

The sacral rhombus of Michaelis is defined by the two posterior superior iliac spines (PSIS), by the spinous process of the fifth lumbar vertebra (L5), and by the upper limit of the inter-gluteal fold, corresponding to the fifth (or fourth) sacral segment (S5). To draw the sacral rhombus: with a dermographic pen, a mark is placed on the skin in a superficial correspondence of the four landmarks described above: right PSIS, left PSIS, spinous process of the fifth lumbar vertebra, and the upper limit point of the intergluteal fold. After that, the rhombus subtended by the four points is drawn and the transverse and longitudinal diagonals. The crossing point of the two diagonals corresponds to the spinous apophysis of the second sacral segment (S2). Between L5 and S2, we have the longitudinal hemi-diameter (Figure 1).

**Figure 2:**
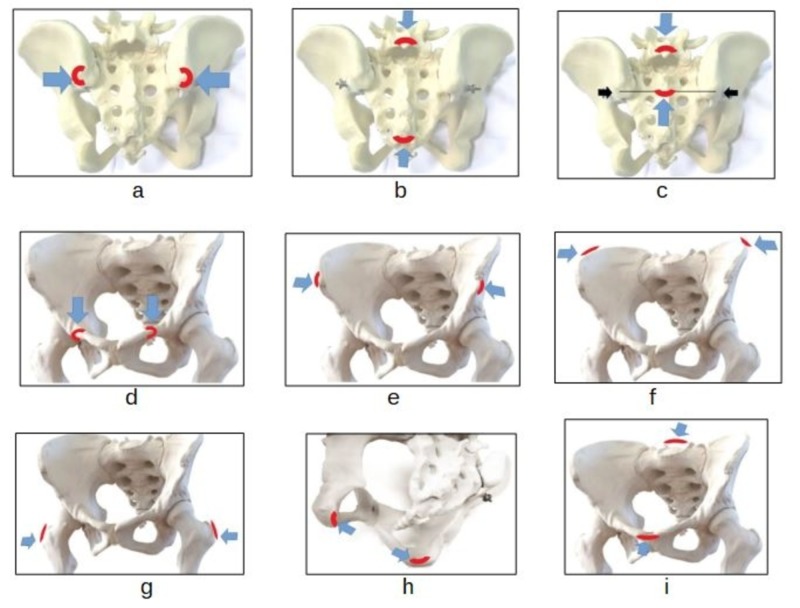
The diameters of the obstetric external pelvimetry for the DEP test a. The transverse diameter of the sacral area b. The longitudinal diameter of the sacral area c. The longitudinal hemi-diameter of the sacral area d. The base of the Trillat's triangle e. The iliac bi-spinous diameter f. The iliac inter-crestal diameter g. The inter-trochanters diameter h. The ischial inter-tuberosities diameter i. The external obstetric conjugate DEP: dynamic external pelvimetry

The transverse diagonal joins the right PSIS and the left PSIS (Figure 2A). The longitudinal diagonal joins the spinous process of L5 and the upper limit of the inter-gluteal fold (Figure 2B) [9,18]. The transverse diameter of Michaelis' sacral rhombus is related to the anterior dimension of the sacral base, which continues the innominate line of the pelvic inlet posteriorly. The longitudinal diameter and the hemi-diameter (Figure 2C) are related to the mobility of the lumbosacral junction (sacral promontory anteriorly).

The Base of Trillat’s Triangle

The Trillat’s triangle is composed of an upper base and two sides. The base corresponds to the superior margin of the pubis and is measured between the pubic insertion of the right and left inguinal ligaments. The sides are the continuation of the inguinal fold on the lower limbs up to the point where they come into contact approaching (subject in the supine position, extended and adducted lower limbs, with light contact between them). The base of the Trillat’s triangle (Figure 2D) would correspond to the transverse diameter of the mid-pelvic area defined between the two ischial spines [9].

The Inter-Tuberous Diameter

The inter-tuberous diameter (Figure 2H) is the transverse diameter of the pelvic outlet; it is precisely the distance between the lower internal margins of the right and left ischial tuberosities, detected in their most caudal and medial point. The operator's fingers are placed close to the angle of insertion of the sacrotuberous ligament.

The Bi-Trochanters Diameter

The bi-trochanters diameter (Figure 2G) is the measurement between the two large right and left femoral trochanters, measured at their most prominent and more cranial outer edge.

The Iliac Inter-Crestal Diameter

The iliac inter-crestal diameter (Figure 2F) is the maximum distance between the lateral margins of the right and left iliac crests measured on the middle axillary line, at the point of their greatest width. It is the upper limit of the region of the iliac fossae (false pelvis).

The Iliac Bi-Spinous Diameter

The iliac bi-spinous diameter (Figure 2E) is the distance between the lateral margins of the anterior superior iliac spines (ASIS). The ASIS can be found cranially at the inguinal ligament and are the anterior limit of the superior margin of the iliac crests. They are palpable as a well-definable. rounded bone margin.

The External Obstetric Conjugate

The external obstetric conjugate (Baudeloque’s diameter) corresponds to the true internal conjugate. It is measured between the outer edge of the upper edge of the pubis and the posterior external point at the first sacral segment (Figure 2I). The anteroposterior distance of the pelvic inlet is between the upper edge of the pubic symphysis and the sacral promontory (the first segment of the sacrum). Often, in the texts, the spinous process L5 is described as being the posterior landmark of the obstetric conjugate, but Baudeloque suggested to position the pelvimeter a little below the fifth lumbar spinous process: that means on the posterior border of the first segment of the sacrum (S1) [18]. The conjugate dynamically tested relates to the sacral nutation and contra-nutation movements [18].

The instruments

The instruments used for the measurement of the external diameters of the pelvis are the Collin pelvimeter and the "bone-meter kit" (BMK) (Metrica SpA, Milan, Italy).

**Figure 3:**
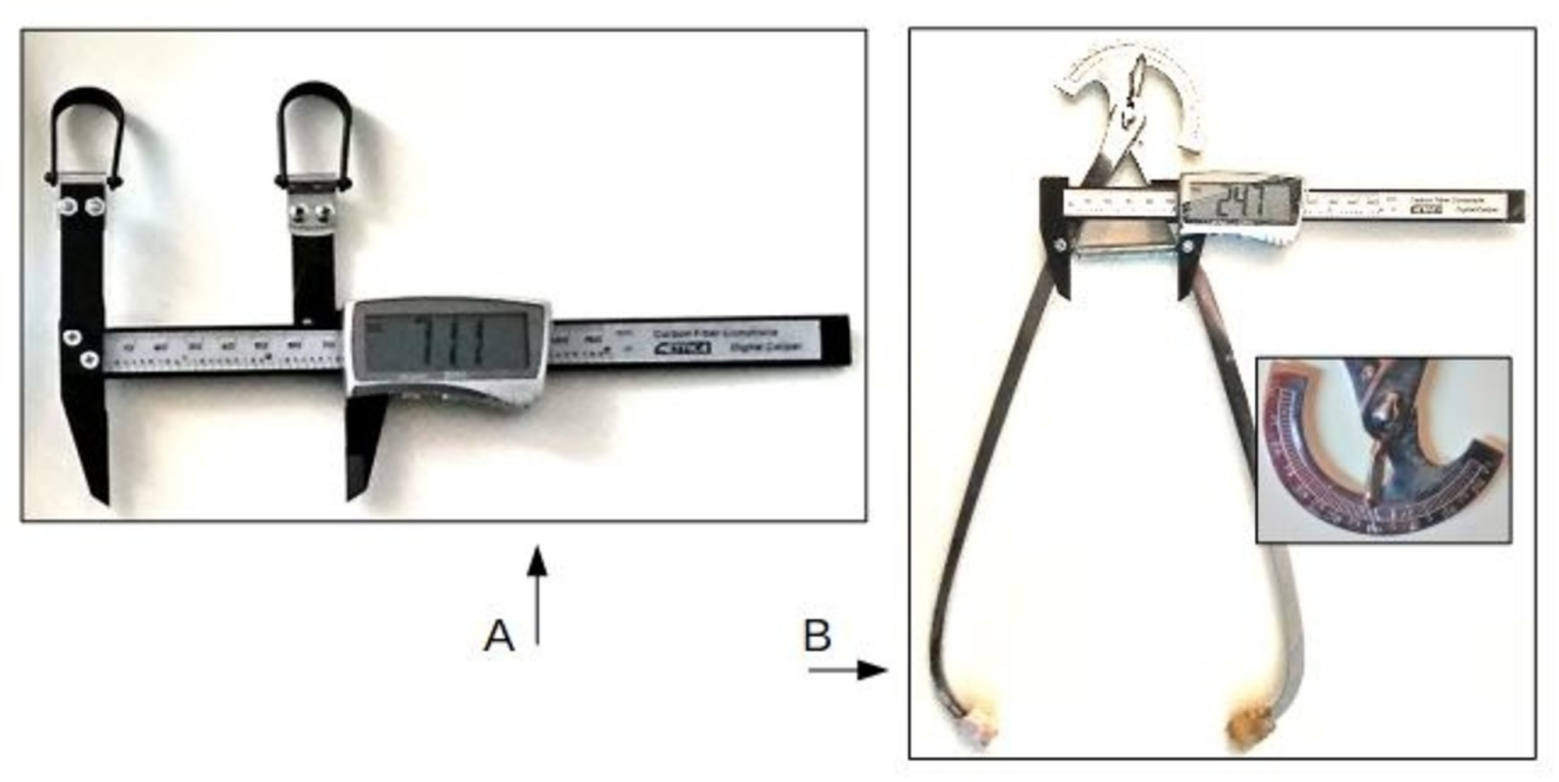
The instrumentation for the DEP test A. The "Bone-Meter Kit" (BMK) by Metrica SpA (Milan. Italy, EU) B. The modified pelvimeter of Collin. Detail of the measurement reading ring. Rubber cups are placed on the extremities of the pelvimeter in order to have softer contact with the skin. DEP: dynamic external pelvimetry

The obstetric pelvimeter used is the Collin pelvimeter, modified to allow stability on the bony landmark, easy handling, and easy and precise reading (Figure 3B). The friction washer, acting as a brake on the fulcrum, has been removed to make the sliding of the two arms free. The arm of the Collin pelvimeter is straight from the fulcrum and curved at the upper end: their distance is 30 cm. Two screws were inserted in the pelvimeter's arms at a distance of 6.5 cm from the lower fulcrum point, for the positioning of the BMK on the anterior side and the spring on the posterior side. A 1.5 N/mm², diameter 0.85 cm and length 6.5 cm spring has been placed on the posterior side, so the pressure force at the upper ends of the pelvis is 1 kg/cm². The spring has the purpose of firmly and steadily adhering the pelvimeter to the bone surface, compacting the superficial soft tissues. The pressure exerted by the springs is always constant and allows the operator to easily manage the pelvimeter. The operator's only attention is to check that the position on the bony landmarks remains fixed while the subject's posture is changed.

**Figure 4:**
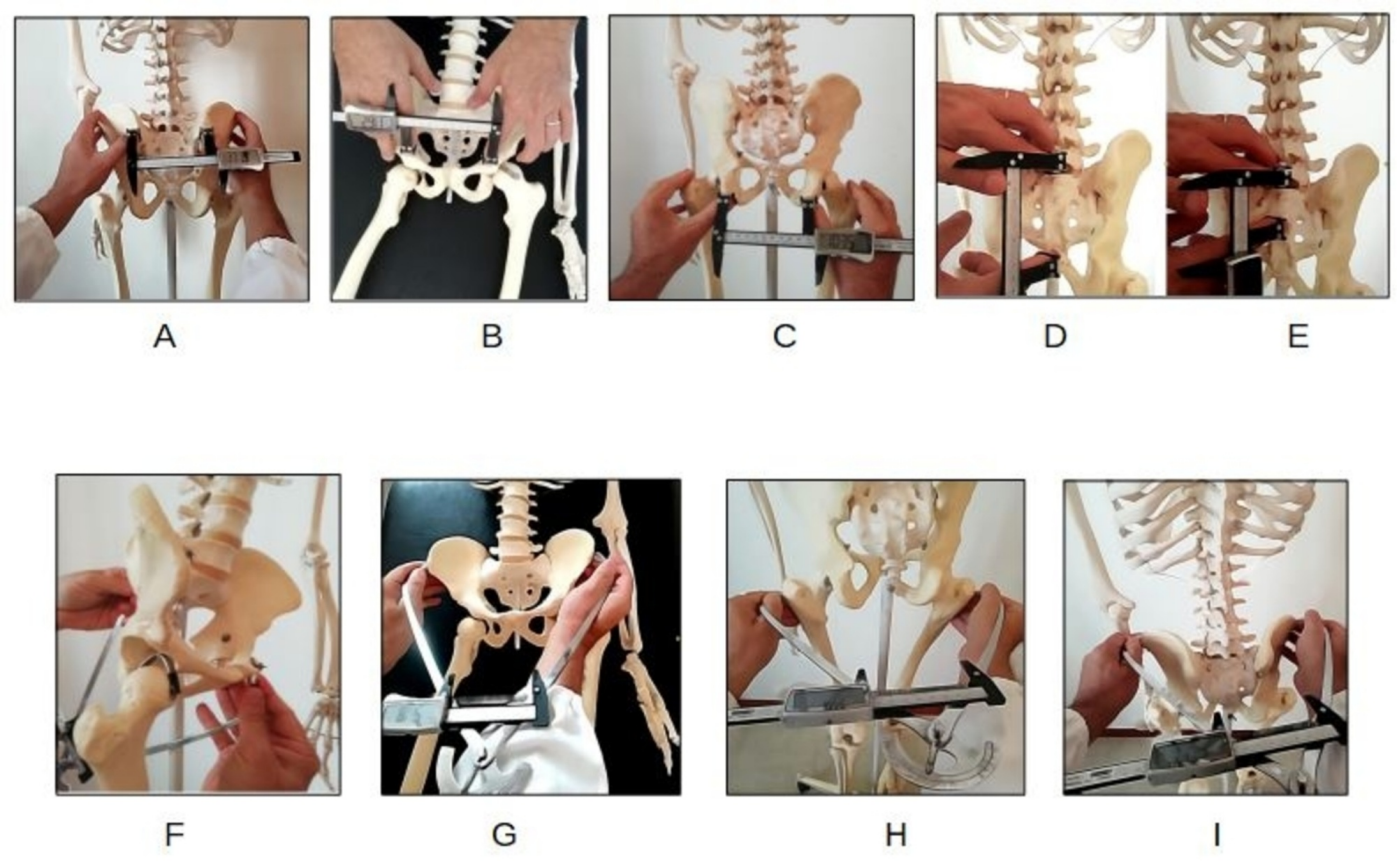
DEP test: handhold of instruments for the measurement of the pelvic diameters Upper row (A-E): external pelvic diameters evaluated by BMK tool. Lower row (F-I): external pelvic diameters evaluated by Collin's pelvimeter A. Michaelis' transverse diameter (transverse pelvic inlet) B. Base of Trillat's triangle (pelvic midlet) C. Inter-tuberous diameter (pelvic outlet) D. Michaelis' longitudinal diameter E. Michaelis' longitudinal hemi-diameter (lumbosacral junction) F. Baudeloque's external conjugate (anteroposterior pelvic inlet) G. Iliac inter-spinous diameter H. Bi-trochanteric diameter I. Inter-crestal diameter DEP: dynamic external pelvimetry

Obstetric pelvimeters have a very uncomfortable and imprecise measurement reading ring (minimum size 5 mm). The BMK has the function of determining the pelvimeter measurement easy, precise, and with a minimum measurement of 0.5 mm. The scale of proportion between the opening of the arms and the measurement detected by the BMK was constructed in a suitable manner using a linear meter certified for anthropometric measurements (Metrica, Milano, Italy). The diameters measurable by the digitized pelvimeter are the bi-trochanters diameter, the inter-crestal diameter, the bi-ASIS diameter, and the external obstetric conjugate (Baudeloque diameter) (Figures 4F-4I).

The BMK is a patented instrument for the digital measurement of anatomical segments. It is a digital reading caliper gauge, with two rings to be worn on the operator's fingers (Figure 3A). The instrument has an accuracy of 0.2 mm and a resolution of 0.1 mm. The operator's fingers wearing the BMK rings remain stable with the bones. The measurement of the distances between the two bony landmarks of each diameter occurs through their direct palpation by the phalanges of the operator's fingers. The operator always keeps the contact firmly and delicately on the anatomical points during the changing positions of the subject to be examined. The accuracy of the instrument does not depend on the operator's tactile and proprioceptive palpatory sensitivity. The operator's tactile and proprioceptive palpatory sensitivity is important to maintain the firmness of the contact on the pelvic landmarks, while the subject changes position.

With the BMK, the following diameters can be measured: the transverse diameter of the sacral area of Michaelis, the longitudinal diameter and hemi-diameter of Michaelis, the base of the Trillat’s triangle, and the bi-tuberous diameter (Figures 4A-4E).

The shifting positions

**Figure 5:**
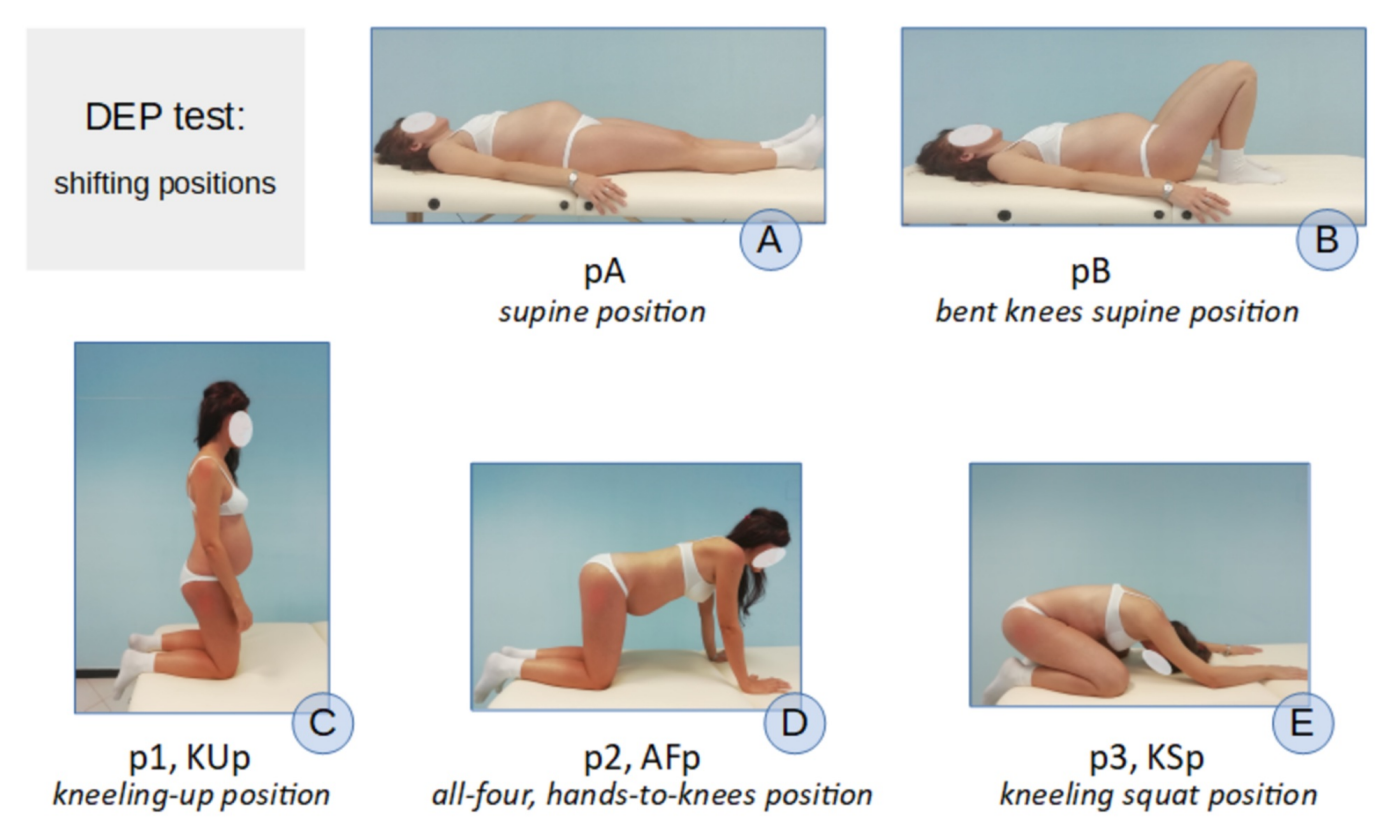
DEP test shifting positions A-B. Supine positions. C-E. Kneeling positions DEP: dynamic external pelvimetry

The different postural patterns used by women during labor and delivery were analyzed, and the results of the instrumental researches that measured internal pelvic diameters in different body positions were considered [1, 4-7,11,13-14]. Afterward, three positions were chosen, differing from each other concerning the degree of flexion-extension of the femoral-acetabular joints and the consequent pelvic rotation (Figure 5):

The three positions were:

- hip extension, in erect position/supine position (Figures 5A-5C);

- hip flexion at 90° (Figures 5B, 5D); and

- maximum hip flexion on the abdomen, unforced, or squat position (Figure 5E).

The 90° hip flexion position is the intermediate position, where the release of the coxofemoral ligaments takes place. The published researches suggest that the greater range of motion, the greater endopelvic diameters can be measured at the intermediate position between the standing position and the squat position [11,13].

On a practical level, the subjects to be dynamically evaluated in their external pelvic diameters will have to assume the following postures, which are indicated if the bony landmarks are posterior, lateral, inferior, and anteroposterior (Figures 5C-5E):

Position one, (p1) "erect position" (Kneeling-Up position, KUp): the subject is upright, neutral, spontaneous, resting on the ground with her knees at hip-width.

Position two, (p2) "hands-and-knees position" (All Four position, AFp): the subject from the position p1 flexes the trunk and the hip joints, bringing support with her hands to the ground [7].

Position three, (p3) "squat position" (Kneeling Squat position, KSp): the subject from the p2 position still flexes the hip joints, bringing the ischial tuberosities as much as possible towards the heels and the head towards the supporting surface [6].

The measurement of the diameters is recorded in position p1, p2, and p3. The operator rests his/her fingers on the bones while the subject is in position p1 and maintains contact up to the position p3 (Video 1).

**Video 1:** DEP test procedure method The video shows the shifting positions (supine and kneeling) to measure the changes in pelvic diameters, the handhold for the BMK, and the practical procedure to perform the Dynamic External Pelvimetry (DEP) test for the base of the Trillat's triangle (corresponding to the midplane of the pelvis) and the transverse diameter of Michaelis' sacral rhomboid area (corresponding to the pelvic inlet). PSIS: the posterior superior ischiatic spine

For the measurement of the anterior diameters (the base of the Trillat’s triangle and the bi-spinous diameter) proceed as follows (Figures 5A-5B). The subject is invited to the supine position with the lower limbs extended and resting on the support surface (position A, pA). When the instrument is placed on the bony landmarks, we ask the subject to bring the heels as close as possible to the ischial tuberosities in order to have flexion of the joints of the hips near 90° (position B, pB): from this position, the feet are allowed to passively slide down, allowing the lower limbs to return to the position A (Video 1). The operator records the measurement of the diameters when the subject is in pB and then in pA. In the supine position, the maximum hip flexion is not achievable for measuring diameters.

The position of the operator is important for the correct positioning of the fingers on the bone landmarks. The position is behind the subject to measure the transversal diameter of the sacral area of Michaelis, the longitudinal diameter/hemi-diameter, the bi-tuberous diameter, the bi-trochanteric diameter, and the inter-crestal diameter. The position is in front, facing the subject's feet, to measure the base of the Trillat’s triangle and the bi-ASIS diameter. The lateral position is used to measure the external obstetric conjugate (Baudeloque’s diameter). Practically, the operator places her/his fingers on the bone landmarks of the diameter of the subject in the starting position registering the measure shown on the screen of the BMK, then closing her/his eyes while the subject shifts the position. The operator opens her/his eyes for reading the measure as the next posture is steadily achieved and so on. The perceptive attention of the operator is firmly and steady upon the bone, not having care of the pelvic general movement below. If any movement is perceived, it means that the contact is superficial on the skin. Closing the eyes allows the beginner operator to be concentrated on the perceptual fingers, avoiding distraction by the measure moving on the screen of the instrument.

Case reports 

As an example, two transverse and longitudinal clinical observational cases are reported. The DEP test highlights the differences between the dynamics of external pelvic diameters in shifting positions in operative and vaginal delivery while the babies' weight (>4000 gr) was similar.

Case Report 1

A nulliparous 34-year-old woman, height 160 cm, with a singleton pregnancy in the cephalic presentation had no health problems before pregnancy, a healthy pregnancy, and pelvic and lumbar pain during the third trimester relieved by osteopathic manual treatments.

The external pelvimetry was performed twice at 32 and 38 weeks’ gestation when the weight was 60 and 62 kg (48 kg before pregnancy: weight gain 14 kg), body mass index (BMI) was 23.4 and 24.2 (+5 and +6). The symphysis-fundus diameter was 30 cm and 39 cm; the baby weight calculated by the Johnson formula was 2635 and 4130 gr. Data from external pelvimetry were reported in Table 1. A difficult operative delivery (vacuum extractor), due to mechanical dystocia, was done at term gestation. A large episiotomy was needed and, consequently, an extensive surgical suture. Baby weight was 4110 gr. She suffered from heavy pelvic-perineal pains and urinary and fecal incontinence, which resulted after delivery. She was relieved from complaints by four months of osteopathic treatments and pelvic yoga exercises. Pain during sexual intercourse was present nine months after delivery.

Case Report 2

A nulliparous 32-year-old woman of height 170 cm, with a singleton pregnancy in the cephalic presentation had a healthy pregnancy, no health problems before, and some light lumbar pain during the third trimester.

External pelvimetry was performed at 38 weeks’ gestation when the weight was 70 kg (55 kg before pregnancy: weight gain 15 kg), BMI 24.2 (+4). Symphysis-fundus diameter 38 cm, baby weight calculated by Johnson formula 3870 gr. Data from external pelvimetry were reported in Table 1. The vaginal natural delivery was at term gestation, intact perineum (small spontaneous superficial lacerations: no need for surgical suture); baby weight was 4100 gr. There were no complaints after delivery.

**Table 1:** DEP test from case reports Data are shown in centimeters (cm) KUp: kneeling-up position. AFp: all-four position. KSp: kneeling squat position. pA: supine position. pB: bent knees supine position *: diameters altered in dystocia, from previous reports and studies DEP: dynamic external pelvimetry

	Case 1 (32w)				Case 1 (36w)				Case 2 (38w)			
Diameter (cm)	Position				Position				Position			
	KUp	AFp		KSp	KUp	AFp		KSp	KUp	AFp		KSp
Sacral transverse*	14.3	15.1		15.1	13.5	13.8		13.9	12.8	13.8		13.9
Sacral hemi-longitudinal	4.3	4.7		4.8	3.3	4.3		4.5	4.8	5.5		5.6
Inter-tuberous*	6.6	8.5		9.5	5.4	6.5		7.6	6.7	7.8		9.8
Inter-crestal	28.4	26.3		25.9	28.9	27.4		27.0	30.7	31.2		30.6
Inter-trochanters	27.6	34.4		37.8	36.0	37.1		40.2	34.4	37.6		39.4
External conjugate*	22,5	23,0		23,5	24,0	23,3		23,0	23,3	23,2		24,1
	pA		pB		pA		pB		pA		pB	
Inter-iliac spines	26,9		26,5		27,4		26,9		29,5		29,1	
Base Trillat’s triangle*	13.9		13.0		11.0		10.6		13.0		11.5	

The babies had the same large weight (4100 and 4110 gr), but the mothers had different heights. In the first case report, the dynamic external pelvimetry test performed at 32 weeks' gestation showed a correct "range of room" (ROR) at the pelvic inlet, midlet, and outlet. Growing the fetus, the pelvic spaces limited their mobility with lumbopelvic discomfort and narrowing as compared with the second case report. The difference (space) between the numbers (the diameter measures in shifting positions), not the rough static measure of the diameter itself is to be considered. The DEP test demonstrated the pelvic diameters’ room change over time, as the baby's growth overcame the maternal pelvic spaces' containing capacity. Pelvic articular mobility is fundamental for daily life movements and childbirth; a physiological ROM had to be safely maintained throughout the pregnancy. The more the baby's weight and size, the less the pelvic tissues can adapt for containing, and the less the range of motion of the external pelvic diameters occurs. The DEP test renews obstetric pelvimetry testing the pelvic movements in a dynamic and postural simulation as close as in childbirth and could be introduced into clinical practice to detect high-risk women for dystocia in labor.

## Discussion

The DEP test is a postural evolution of traditional external pelvimetry and was designed as a clinical screening approach to pregnancy and childbirth. A preliminary clinical report presented at the “13th World Congress of Perinatal Medicine” [19] described the results from 364 pregnancies' data, showing a true positive rate of 0.98, a true negative rate of 0.81, a false negative rate of 0.01, just considering the transverse diameter of Michaelis' sacral area, and cut-off at the 15° percentiles. The strength of the test is that it can be performed in any obstetric clinic around the world, it is safe to execute for pregnant women, and easy for operators. It could be introduced into the clinical routine in all pregnant women: mechanical dystocia is a missed diagnosis in low-risk pregnancy and primiparas.

During pregnancy, the pelvic diameters change with the fascial and ligamentous quality of the pelvis and the spine, subject to the action of relaxin, and probably the increase in uterine and body size requires an increase in the pelvic diameters [1]. The pelvic diameters are related to the following joints: the lumbar-iliac, lumbosacral, and sacrococcygeal junctions; the sacroiliac, femoral-acetabular, and pubic symphysis joints. The range of external and internal pelvic diameters depends on the range of motion (ROM) of the joints of the pelvis, that is related to the tension state of muscular, ligamentous, and fascial balance: lumbopelvic pain in pregnancy predisposes to dystocia from such altered balance tension [10]. Pain is part of the inflammatory process related to a limited excursion of the pelvic joints movement and the dense/tense quality of the soft tissues. Consequently, the internal spaces of the pelvis could be mechanically altered impairing the biodynamics of the birth canal and birth process.

The postures to be used for the DEP test are easy and comfortable to perform and maintain by all women at full-term pregnancy, by untrained or overweight women, by those who suffer from musculoskeletal pains or physical complaints. The diameters in the standing and kneeling erect position are stackable (personal unpublished data). The hands-and-knees and the semi-squat position are among the positions used by women and encouraged by midwives during labor and delivery [5-7]. The standing squat position is a hard challenge for the greater number of pregnant women, and the feet position during the standing squat position can negatively affect the pelvic diameters [6]. The kneeling squat position avoids these biases, it is easy to achieve and maintain by all the women, and it is well sustained by MR studies that show the more room in the pelvis from the supine and standing positions [11,13]. The kneeling and supine postures fulfill the needs of both operators and subjects to be safe and comfortable during the diagnostic technique; movements are non-exhausting to be repeated several times.

The instrument must remain in constant, stable, and firm contact with the bony landmarks during the transition from one posture to the next: constant contact from the first to the last measurement could give to the technique reliability and precision. Several studies showed that the poor reliability of clinical tests involving palpation may be explained by errors in the landmark location. Recognizing the anatomical bone points of the pelvis and finding them constantly over time are among the possible biases of the method [20]. Dynamic pelvimetry refers to the ROM, evaluating the difference between two measurements obtained without moving the firm contact on the bone landmarks. The difference between the measurements is independent of the exact point where the instrument is positioned while the static diameter evaluation shows poor reliability. The inaccuracy of the manual palpation of the bony landmarks can be overcome considering the delta, the difference between the measurements obtained in different positions: the range of motion is independent of the exact point of measurement. The instrumentation has been designed and constructed so that the operator can easily maintain a stable and firm contact on the bone landmarks during the subject's movements in shifting the position.

Our previous preliminary report [19] and the published studies [6-9,11] seem to confirm the greater importance of some pelvic diameters as compared to others in dystocia, and the largest diameter is measured in the intermediate position (hands-and-knees position). Further studies need to provide the reproducibility and the reliability of the test and its effectiveness for the screening of mechanical dystocia and “contracted pelvis.”

## Conclusions

The DEP test is a dynamic biomechanical measurement method to evaluate the "range of room" of the obstetric external pelvic diameters in shifting positions. It is adherent to the reality of the positions that women adopt to manage labor and delivery. It is simple to put into practice and could be a method that contributes to the prevention of mechanical dystocia and its consequences during childbirth. A shared method is a first step to carry out large-scale homogeneous clinical studies.
